# Targeted memory reactivation during sleep boosts intentional forgetting of spatial locations

**DOI:** 10.1038/s41598-020-59019-x

**Published:** 2020-02-11

**Authors:** Eitan Schechtman, Sarah Witkowski, Anna Lampe, Brianna J. Wilson, Ken A. Paller

**Affiliations:** Department of Psychology, Northwestern University, Evanston, IL 60208 USA

**Keywords:** Non-REM sleep, Replay, Consolidation, Forgetting

## Abstract

Although we experience thousands of distinct events on a daily basis, relatively few are committed to memory. The human capacity to intentionally control which events will be remembered has been demonstrated using learning procedures with instructions to purposely avoid committing specific items to memory. In this study, we used a variant of the item-based directed-forgetting procedure and instructed participants to memorize the location of some images but not others on a grid. These instructions were conveyed using a set of auditory cues. Then, during an afternoon nap, we unobtrusively presented a cue that was used to instruct participant to avoid committing the locations of some images to memory. After sleep, memory was worse for to-be-forgotten image locations associated with the presented sound relative to those associated with a sound that was not presented during sleep. We conclude that memory processing during sleep can serve not only to secure memory storage but also to weaken it. Given that intentional suppression may be used to weaken unpleasant memories, such sleep-based strategies may help accelerate treatments for memory-related disorders such as post-traumatic stress disorder.

## Introduction

Out of the multitude of events humans experience each day, relatively few are retained as declarative memories to support subsequent recall or recognition. Whereas forgetting has often been viewed as a negative, its adaptive role has gained prominence^1^. Additionally, control over memory processes, including the retrieval of intrusive memories, has been hypothesized to support emotional regulation^2^. Deficits in the capacity to actively suppress intrusive memories has been associated with disorders such as post-traumatic stress disorder^3^.

Forgetting involves the passive decay of memories^4,5^, but inhibitory neurocognitive mechanisms may also contribute to declining recollective abilities. This effect, termed active forgetting, is supported by adaptive, flexible processes that suppress memory-related brain networks^6–8^. Possible mechanisms for active forgetting range from prefrontal inhibition^6^ on the systems level to dopaminergic forgetting cells^7^ and neurogenesis^8^ on the cellular level. Most of these models are similar in that they involve a newly acquired, learning-like process that targets core memory structures or cells and suppresses them. An outstanding question is whether these inhibitory circuits, established through suppression learning, are strengthened during sleep, as is the case for declarative and nondeclarative memories generally^9^.

Several memory paradigms, such as extinction and “Think-No Think”^10^, have attempted to model active forgetting by using intentional, motivated suppression of memories. Another paradigm, item-based directed forgetting, involves exposure to remember- or forget-instructions directly after exposure to an item. To-be-forgotten (TBF) items are later recalled at lower rates relative to to-be-remembered (TBR) items^11^, an effect putatively mediated, in part, by active inhibitory processes^12^. Accounts of directed forgetting commonly invoke differential encoding between TBF and TBR items as well as retrieval inhibition^13^, whereas consolidation has rarely been addressed in the context of directed forgetting.

In this study, we sought to consider whether suppression of spatial memory that was established during wake can be selectively boosted during sleep. Towards this goal, we used a sleep intervention termed targeted memory reactivation (TMR). In auditory TMR designs, sleeping participants are exposed to sounds that were previously associated with specific learning episodes^14^. These cues are typically presented unobtrusively during non-REM (Rapid-Eye-Movement) sleep and have been shown to improve performance on visuospatial and procedural memory tasks and improve vocabulary and word-pair learning^15–18^. If inhibitory memory-suppression mechanisms are adaptively learned, TMR could potentially enhance wake-related forgetting effects in an item-specific manner. Indeed, several studies have attempted to employ TMR to enhance fear extinction, producing conflicting results (see^19^ for review).

More recently, cues related to memory suppression were presented during sleep and found to decrease the strength of other memories formed in a separate task^20^. In a first demonstration of targeted forgetting during sleep, Simon and colleagues^20^ employed TMR to induce *de-novo* forgetting using the directed-forgetting task. Participants first associated a sound with the instruction to forget in a word-learning directed forgetting task. Next, they viewed a set of objects, each in one of four screen locations, and a corresponding sound was presented with each object. The forget cue was presented during slow-wave sleep (SWS) with the sounds of five of the objects. One week later, participants showed a significant decrease in recall for these objects relative to non-cued objects. This study was the first to show an effect of TMR on declarative forgetting. The authors suggested that the forget command was activated during sleep, and its novel association with the five object-related cues induced forgetting of those objects.

However, these results could have stemmed from the conjoint sleep-related activation of information from two unrelated tasks and not from the specific effect of the cue for forgetting. In other words, the activated memory traces may have interfered with one another, indirectly inducing a forgetting effect. Whereas it is established that temporally proximate cues disrupt TMR-induced benefits^21,22^, the conditions under which temporally adjacent memories may harmoniously interact – but not interfere – during sleep are not known.

**Figure 1:**
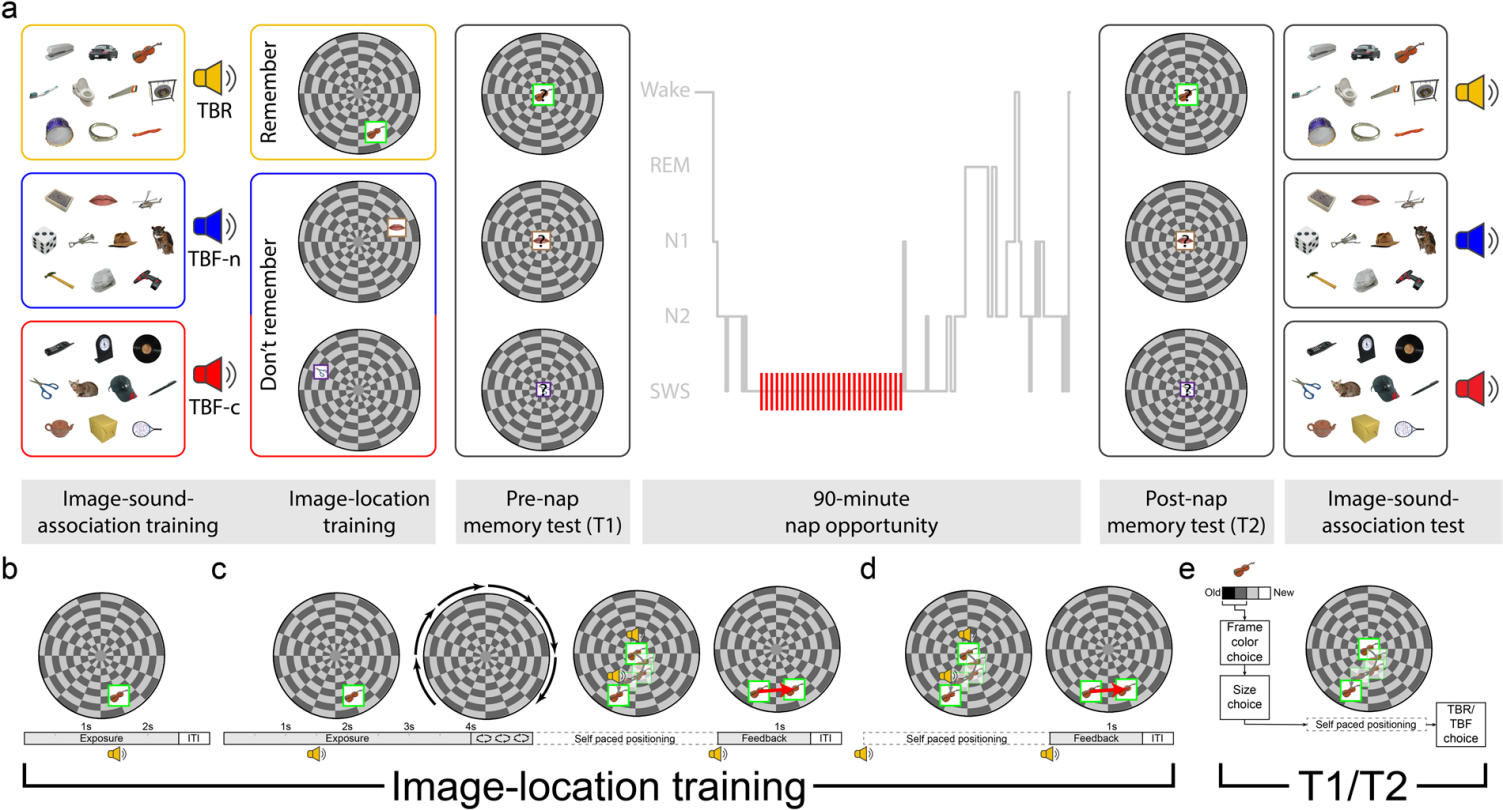
Experimental design. Participants first learned to associate three sets of ten images with three corresponding sounds. Next, each sound was associated with instructions to either remember the location, along with other features of the image (to-be-remembered, TBR), or not commit them to memory (to-be-forgotten, TBF). In the following image-location training stage, each image was presented in a random location on a circular grid. This was followed by a pre-nap memory test, in which image memory for these features was tested for all items (participants were explicitly instructed to try their best even for TBF items). During NREM in the subsequent 90-minute nap opportunity, participants were presented with the TBF-c (to-be-forgotten – cued) sound, but not the TBF-n (to-be-forgotten – not-cued) sound or the TBR sound, at unobtrusive levels. After waking up, the participants were again tested on their memories for both image-location and image-sound associations. The hypnogram shows actual data for one of the participants. Images presented in this figure are similar to the ones used in the experiment itself, were obtained from the Bank of Standardized Stimuli (BOSS; https://sites.google.com/site/bosstimuli)^38^ and are licensed under the terms of Creative Commons Attribution-Share Alike 3.0 license (http://creativecommons.org/licenses/by-sa/3.0/).

Our study can be considered a conceptual replication of the study conducted by Simon and colleagues^20^. However, the focus of our study is on whether effects of memory suppression during wake can be enhanced during sleep. We used TMR with forgetting cues to investigate forgetting of the spatial location of TBF items. Participants first learned to associate three sounds with three groups of images, respectively, in a visuospatial task. As shown schematically in Fig. 1, two of these sounds were then associated with the instruction to forget features of the images, including their location on a circular grid, and the third with the instruction to remember. During non-REM sleep, one of the two sounds associated with the forgetting instruction was presented (cued sound; TBF-c). We hypothesized that spatial recall would be worse for the associated images than for the remaining TBF images (TBF-not cued; TBF-n). By contrasting image groups associated with identical instructions before sleep, this manipulation allowed us to isolate the TMR effect on forgetting. Worse spatial recall in the TBF-c condition than in the TBF-n condition would imply that active forgetting can be enhanced during offline periods of sleep.

## Results

**Figure 2:**
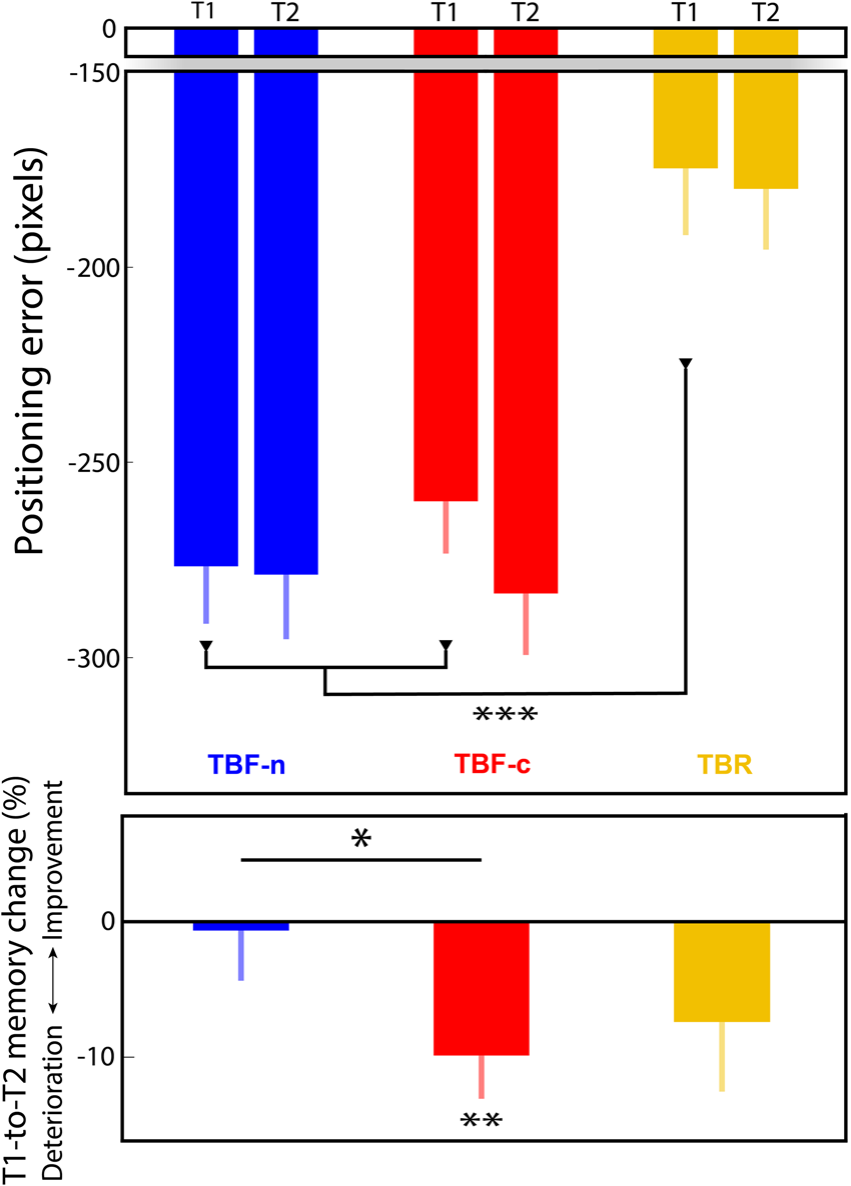
Sleep was detrimental to spatial memory selectively for TBF-c images. Top – absolute positioning errors for TBF-n (blue), TBF-c (red) and TBR (yellow) images before sleep (T1) and after sleep (T2). Zero error represents exact positioning. Significant difference between errors in T1 and T2 errors are marked in the bottom panel. Bottom – difference between positioning errors in T2 and T1 computed as a percentage change. Negative values represent memory deterioration over sleep. Error bars represent standard errors of the mean. ***p < 0.001, **p < 0.01, *p < 0.05.

Tables 1 and 2 show the sleep architecture and frequency of cue presentation during sleep, respectively. Localization errors for TBR, TBF-c, and TBF-n conditions for pre- and post-sleep tests (T1 and T2, respectively) are shown in Fig. 2 (top). As a manipulation check, we first compared spatial memory for all TBF images (i.e., collapsing across TBF-c and TBF-n) versus TBR images. Using a 2 × 2 repeated-measures ANOVA, we found that spatial memory error rates for TBF images were higher than those for TBR images, consistent with more forgetting when instructions were to forget [*F*(1,29) = 44.73, *p* = 2*10^−7^]. There were no significant differences between pre- and post-sleep results [*F*(1,29) = 3.34, *p* = 0.08], nor was there a significant interaction [*F*(1,29) = 0.37, *p* = 0.55], providing no evidence for a selective impact of sleep (or delay) for TBR versus TBF.

**Table 1:** Sleep architecture (mean ± SEM).

	Total time with lights off	Wake	N1	N2	N3	REM
Minutes	91.92 ± 1.14	21.57 ± 3.59	12.47 ± 1.76	24.25 ± 2.54	31.37 ± 3.34	2.27 ± 0.85
Percentage	100%	23.29 ± 3.86	13.42 ± 1.78	26.37 ± 2.71	34.49 ± 3.7	2.43 ± 0.91

**Table 2:** Cuing during sleep (mean ± SEM).

	Total	Wake	N1	N2	N3	REM
Number of cues	236.87 ± 21.88	2.8 ± 1.68	4.73 ± 3.1	34.63 ± 11.63	194.7 ± 25.54	0 ± 0

Our main hypothesis concerns the two TBF conditions. Therefore, we next focused on differences between TBF-n and TBF-c. The pre-sleep instructions for these two conditions were identical. Therefore, as expected, sound-item associations and training patterns were similar for TBF-c and TBF-n conditions (Supplementary Fig. 1). Additionally, errors at T1 (i.e., before sleep and TMR) were not significantly different [*t*(29) = 1.08, *p* = 0.29]. Participants were also asked before sleep whether items were associated with TBR or TBF instructions. There were no differences in accuracy rates between TBF-c and TBF-n items [$\bar{x}=94.45\pm 1.77;\bar{x}=94.4\pm 1.51$ respectively; *t*(29) = 0.02, *p* = 0.98].

We considered the change in memory over sleep by calculating the percentage of change in spatial error for each condition. TBF-c items significantly deteriorated between T1 and T2 [*t*(29) = −3.04, *p* = 0.005; Fig. 2B], whereas TBF-n and TBR items showed no significant change [*t*(29) = −0.17, *p* = 0.86; *t*(29) = −1.74, *p* = 0.09, respectively]. Our main hypothesis was that the memory deterioration over sleep would be larger for the TBF-c group relative to the TBF-n group. The results supported this hypothesis, as a direct comparison of memory change in the two TBF conditions showed a larger detrimental effect of TMR on spatial memory for the TBF-c condition relative to the TBF-n condition [*t*(29) = −1.74, *p* = 0.046]. However, considering the change (in percentage) in all three conditions (TBR, TBF-c, and TBF-n) using a repeated-measures ANOVA did not yield a significant difference between groups [*F*(2,29) = 1.21, *p* = 0.30].

Whereas there was more forgetting across sleep in the TBF-c condition than in the TBF-n condition, as predicted, there were also small differences at baseline. Yet, the pre-sleep error rates for TBF-c and TBF-n conditions were not significantly different. Still, we sought further confirmation that small differences in baseline error rates did not artifactually contaminate the results. We created 10^5^ subsampled datasets in which data for a single TBF-n and a single TBF-c image was randomly omitted for each participant. Out of these datasets, we chose only the ones in which the significance of the pre-sleep effect was *p* > 0.5 (Supplementary Fig. 2, top). For these subsampled sets, selected for their comparable pre-sleep error rates, the mean significance rate for the detrimental TMR effect was 0.11 (median = 0.095), indicating a tendency towards significance even when compensating for random pre-sleep differences.

**Figure 3:**
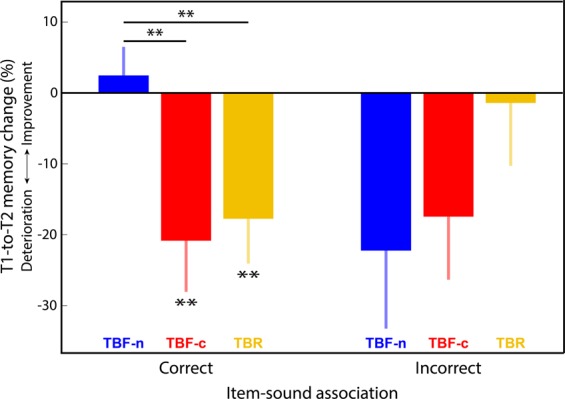
The detrimental effect of TMR was limited to the items correctly associated with the sounds. Bars show the percentage change from T1 to T2 for TBF-n (blue), TBF-c (red), and TBR (yellow) images. Negative values represent memory deterioration over sleep. Bars at left show results for images with correct sound associations; bars at right show results for images without correct sound associations. Error bars represent standard errors of the mean. **p < 0.01.

A possible weakness in our design is that any effect of cuing is contingent not only on a strong association between the presented sound and the instruction to forget, but also on strong links between the sound and the ten individual cued items for each sound. To insure this was the case, participants trained extensively on forming these links before the nap, although performance was not perfect. The post-nap recall test revealed that the average number of images correctly recalled as associated with the sounds was 7.07 for the TBR sound and 5.78 for the two TBF sounds (median 7 and 5.5, respectively). We therefore repeated the analyses conducted for the full dataset (Fig. 2), limiting them to images correctly recalled. As with the full dataset, error rates for TBF images were higher than those for TBR images [*F*(1,29) = 39.49, *p* = 7*10^−7^]. However, unlike the full dataset, there was a significant difference between pre-sleep and post-sleep results [*F*(1,29) = 4.65, *p* = 0.04], indicating a general detriment in memory between T1 and T2. The interaction remained nonsignificant [*F*(1,29) = 0.02, *p* = 0.88], indicating that there was no evidence for a selective impact of sleep (or delay) for TBR versus TBF. Importantly, as shown in Fig. 3 (left), memory significantly deteriorated between T1 and T2 for correctly recalled TBF-c items [*t*(29) = −2.84, *p* = 0.008] but not TBF-n items [*t*(29) = −0.61, *p* = 0.55]. The error rate for the TBR items was also higher for T2 relative to T1 [*t*(29) = −2.76, *p* = 0.01]. Confirming the main finding from the full dataset, the detrimental effect of TMR on spatial memory was significant when considering only the recalled items [i.e., the detrimental TMR effect for TBF-c was stronger than for TBF-n; *t*(29) = −2.72, *p* = 0.005]. Additionally, the repeated-measures ANOVA including all three conditions (TBR, TBF-c, and TBF-n), yielded a significant difference between groups [*F*(2,29) = 3.9, *p* = 0.026].

In this analysis limited to images correctly recalled in relation to the sound, there is again the possible influence of pre-sleep error rates, which were marginally different between TBF-c and TBF-n for this dataset [*t*(29) = 2.01, *p* = 0.054]. To rule out this possibility, we used the previously described subsampling method. Accordingly, when pre-sleep differences were minimized (significance level of *p* > 0.5), the average significant rate for the cuing effect (i.e., TBF-c vs. TBF-n) was 0.05 (median = 0.037), indicating a strong tendency towards significance (Supplementary Fig. 2, bottom). We therefore conclude that the TMR effect that was observed did not result from pre-sleep differences.

Finally, for the sake of completeness, we considered the effect of cuing for items that were not exclusively associated with their correct sound. Since these items were not strongly linked with the correct sound, we expected that the TMR effect from presented the TBF-c sound would be substantially reduced. For this dataset, both the TBF-c and TBF-n item groups showed a trend towards higher error rates after sleep [*t*(29) = −1.93, *p* = 0.06; *t*(29) = 1.99, *p* = 0.06, respectively; Fig. 3, right], but errors for these groups were not significantly different from one another [*t*(29) = 0.31, *p* = 0.76]. The error rate for the TBR items was not significantly different between T2 and T1 [*t*(26) = −0.15, *p* = 0.88; fewer degrees of freedom due to all items successfully recalled for some items]. The repeated-measures ANOVA including all three conditions did not yield a significant difference between groups [*F*(2,26) = 1.18, *p* = 0.32]. Therefore, when sound associations were not well learned, memory was apparently not affected by TMR.

The detrimental effect of cuing (i.e., the TBF-c – TBF-n difference) was not correlated with the number of cues presented during NREM [*r* = −0.27, *p* = 0.14 for full dataset; *r* = 0.09, *p* = 0.65 for correctly recalled] or SWS [*r* = −0.31, *p* = 0.1 for full dataset; *r* = 0.08, *p* = 0.68 for correctly recalled]. There were also no significant correlations with the percentage of time spent in NREM [*r* = −0.3, *p* = 0.11 for full dataset; *r* = 0.06, *p* = 0.77 for correctly recalled] or SWS [*r* = −0.24, *p* = 0.2 for full dataset; *r* = 0.02, *p* = 0.92 for correctly recalled].

## Discussion

In this study, we showed that presenting suppression-related auditory cues during an afternoon nap selectively weakened spatial memory for associated images. As part of a visuospatial task inspired by previous studies of directed forgetting, each of two arbitrary sounds was first associated with (1) a set of images, and then with (2) instructions to avoid committing the features of these images to memory. Participants were instructed to remember other images, and their spatial memory performance was indeed better for these images relative to the TBF images. The two critical forgetting conditions were closely matched in many ways: images were randomly assigned to each condition in each participant, both sounds were associated with identical instructions to forget, and the specific sound associated with each condition was counterbalanced across participants. One of these sounds (TBF-c) but not the other (TBF-n) was presented during sleep. Based on post-sleep spatial recall results, we deduce that cue presentations during sleep had a detrimental effect on associated memories. Whereas not all images were successfully linked with the sound, greater forgetting for TBF-c than TBF-n images was observed only for those that were.

In prior TMR studies, cues during sleep have generally improved recall^19,23^, whereas here the cues apparently reactivated memory-suppression instructions so as to produce a decline in recall accuracy. These results are in line with those from another study that used TMR to induce forgetting based on a different directed-forgetting procedure^20^. Taken together, our studies provide compelling evidence that manipulation of memory processing during sleep may be used to weaken memories in certain contexts. The two experimental designs differ from each other in several important respects. First, the directed-forgetting sound used by Simon and colleagues gained its meaning in the context of one memory task and the critical memories reactivated during sleep came from another task. Also, the design used by Simon and colleagues^20^ could not rule out the possibility that this sound influenced memory storage due to general memory interference. In other words, forgetting may have arisen due to a specific action of the cue or due to a generically disruptive effect of a sound from a different memory task. Further data are needed to discriminate between these two alternatives. Another important difference between the two studies concerns the context in which stimuli were learned. In our study, forgetting was an integral part of the pre-sleep task, whereas in the Simon *et al*. study the sounds during sleep acted to transfer the forgetting instruction to item-location memories. In that sense, the design of Simon *et al*. included a new association formed during sleep, akin to sleep studies that introduce new learning such as conditioning^24^.

Despite these differences between the studies, our study serves as a conceptual replication of the previous one. Together, the accumulated data are helpful in suggesting a possible mechanism by which TMR enhances forgetting. In our study, the sound cue was associated with both the ten specific images and the instruction to forget. In directed forgetting, as in other experimental designs that include forgetting and memory suppression (e.g.^25^), there are specific actions associated with instructions to forget. These actions could concern encoding strategies (e.g., diverting attention away from an item) or intentional suppression of already acquired memories. Our data can be explained by assuming that TBF-c sounds presented during sleep reactivated both memories for the associated images and memories for forgetting actions. Simon *et al*.^20^ presented a generic forget cue and an item cue conjointly, rather than using a TMR cue with a pre-established association with specific items. The two studies thus used different procedures with different limitations, but together the results provide strong support for the idea that an active, forgetting action can be engaged using TMR.

Although the focus of our study was on forgetting, it was also one of the first to use a single auditory cue to activate memories for multiple items during sleep^26–28^. Previous auditory TMR studies have predominantly used congruent sounds for cuing (e.g., cat – meow), whereas our study is the first to use a large set of items linked with a random auditory cue. Interestingly, our results suggest that the suppression effect is contingent upon the explicit sound-image associations. Future studies should consider whether this result is generalizable to other forms of TMR-enhanced learning when the typical goal is to improve memory. Olfactory TMR designs have predominantly used arbitrary odors^29,30^, but to the best of our knowledge the role of explicit knowledge regarding individual odor-item associations has not been directly considered.

Previous findings regarding the contribution of sleep to directed forgetting have been inconclusive. Whereas one study found worse memory for TBF words with a retention interval that included sleep relative to wake^31^, the more common finding is better memory for TBR items but not worse memory for TBF items^32–34^. Despite the many similarities to classic directed-forgetting designs, two key differences make it difficult to compare the sleep effects between our design and previous ones. First, directed forgetting tasks commonly employ individual words that participants commit or do not commit to memory. Recollection is then evaluated using recall or recognition tests, and words are scored as either remembered or forgotten. Our task used far fewer items than in typical directed-forgetting experiments. Additionally, in our task, we did not test recall of words, but rather different features of images, using non-binary scales such as a location on a continuous 2-D grid. Previous studies in our lab using the same spatial-memory task have found that memory is worse post-sleep relative to pre-sleep, although sleep still protects memory relative to an equivalent period of time awake^15,35^.

Another difference is that in our design, participants were exposed to each image multiple times, with active manipulation of the image. In classic directed-forgetting tasks (e.g.^12^), each word is shown only once, followed by the TBR/TBF cue. This difference is important because it means that the level of initial learning, which is a crucial aspect of directed-forgetting paradigms, may have been higher in our task relative to that in typical designs. Additionally, by the second exposure and certainly further on, participants may have known that an image was a TBF item before the cue was explicitly presented, and that knowledge may have had an impact on the level of attention dedicated to that image and its features.

It is now widely accepted that TMR can be used to enhance memories and reduce forgetting^14,19^. The exciting possibility of decreasing the strength of memories over sleep introduces new questions about both memory and sleep. It further established the notion that at least some forms of forgetting are achieved using an active process that involves inhibitory learning. It is too early to say whether every form of memory suppression may benefit from TMR, but the possible implications are nonetheless exciting. Besides using TMR to suppress everyday unwanted memories, it may be used to enhance and accelerate treatments for anxiety disorders and post-traumatic stress disorders. These potential benefits may lead to an improvement in the quality of life of millions of people who suffer from such disorders. Future research should therefore aim to expand our understanding of the boundary conditions and possible applications of TMR to enhance forgetting.

## Methods

### Participants

Participants were members of the Northwestern community with no known history of neurological or sleep disorders who claimed to be able to nap in the afternoon. The study was first run on 33 participants, and later an additional 11 participants were recruited and run on the exact same protocol following a reviewers’ concerns regarding statistical power, bringing the total sample size to 44. Participants were asked to go to bed later than usual the night before the study, wake up one hour earlier in the morning, and avoid any caffeine on the day of the study. Results exclude data from 14 of the 44 participants. Three participants were not sufficiently cued during sleep. One participant’s data was not collected due to technical problems. Ten other participants had poor results in an image-sound-association recall test, correctly associating less than three images out of ten per sound. The rationale behind the exclusion of this latter group is that our task used arbitrary (i.e., non-congruent) sound-image associations such that TMR is contingent on forming strong associations between each sound and the corresponding images. The final sample (*N* = 30) included 21 females and one non-binary person with ages between 18 and 34 yrs (mean ± SD = 21.63 ± 4.21). The Northwestern University Institutional Review Board approved the procedure. All methods were performed in accordance with the relevant guidelines and regulations and informed consent was obtained from all participants.

### Materials

Stimuli for this task consisted of images of single objects, people, or scenes. Out of a total of 80 color images (e.g., a car, a woman laughing, a basketball), 30 images were randomly selected per participant and divided into three groups of ten. Each group was randomly associated with one of three sounds. The sounds, which were 500 ms in duration, were not semantically related to any particular image and were distinguishable from each other. One of the sounds was associated with the TBR instruction for all participants. The other two sounds were assigned to one of two TBF conditions according to an arbitrary rule (i.e., whether the participant number was odd or even). As described below, one of these two sounds was presented during sleep. These conditions will be referred to as TBR, TBF-c (to be forgotten - cued) and TBF-n (to be forgotten – not cued). When referring to the last two groups collectively, they will be termed TBF. Our decision to limit each group to only ten items, limiting statistical power, was intentional and originated from the need to produce an appropriate level of task difficulty and enable participants to learn to associate images with their arbitrary sound. Each image was randomly assigned one of eight distinct frame colors and a size ranging between 50 and 100 pixels. Additionally, each image was positioned in a specific location on a circular grid with a radius of 540 pixels (135 mm), under the constraints that its center was at least 50 pixels from the middle and at least 100 pixels from the border of the circle. In addition to the 30 images associated with the sounds, two additional sets of ten images were used as novel images.

Visual stimuli were presented on a 1920 × 1080 pixel screen (P2217Hb, Dell Inc., TX). Sounds were delivered using PC speakers (AX-210, Dell Inc., TX). Stimulus presentation was controlled by Neurobs Presentation (v17.2). Scripts and stimuli are available upon request.

### Procedure

Participants arrived at the lab between noon and 2:00 pm. After consenting to participate in the study, they entered an experimental chamber and started the task.

In the first phase of the study (“Image-sound-association training” in Fig. 1a), participants were instructed to learn to associate each of the 30 images with one of three sounds (i.e., 10 images were randomly associated with each sound). Importantly, this was done before they were informed which sound would later cue forgetting and even that the study included instructed forgetting. Participants first viewed each image in a random order in the middle of the screen for 2500 ms, followed by a 1500-ms white screen during the inter-trial interval (ITI). The sound for each image was presented twice: coinciding with the image presentation and co-terminating with it. After all 30 images were presented once, participants viewed a screen including all 30 images and heard one of the three sounds. They were asked to select, using the mouse, the ten images associated with the sound. Participants could choose to hear the sound again. After choosing up to ten images, the correct answers were revealed and participants could use this feedback to improve their memory for the image-sound associations. Both the selection and the feedback stages were self-paced. This test was repeated for each of the three sounds in random order. This testing phase continued until the participant identified at least eight images correctly per sound or tried the test five times.

In the second phase of the study (“image-location training” in Fig. 1a, presented in detail in Fig. 1b–d), participants were informed that they would be exposed to the images and asked to encode their features, including the image size, frame color, and location on a circular grid. Crucially, however, they were instructed to only memorize features for images presented with one sound and not commit to memory any features of the images associated with the other two sounds. A pilot experiment conducted before data collection revealed location was most affected by these directed-forgetting instructions, whereas other features did not show significant differences (Supplementary Fig. 3). We decided before running the study that we would use the data on location recall to test our hypotheses about forgetting, not the other memory measures. We thus avoided the need for corrections for multiple comparisons that might be needed if all memory testing was pertinent (but see Supplementary Fig. 3 for post-hoc analyses). We reasoned that removing non-spatial learning requirements of the task such as frame color and image size might have reduced the cognitive load and indirectly affected forgetting for locations. We therefore decided, ahead of data collection, to focus on spatial memory but keep the task similar to the one piloted.

Image-location training consisted of three sequential stages. In the first stage (Fig. 1b), each image appeared on the grid in its designated location and 1500 ms later the associated sound was presented. Another 1000 ms later, a white screen appeared and a 500-ms inter-trial interval commenced. As noted, the instructions during this stage and subsequent stages indicated that participants should either commit the item features (location, frame color, and size) to memory or avoid doing so, depending on the presented sound. A total of 60 trials were presented in this stage, two for each image (order pseudorandomized to avoid consecutive repetitions of the same image).

After this stage, participants were given the opportunity to refresh their memories regarding which sound went with which instruction by clicking one of three boxes (one per sound) in a self-paced manner. No images were presented during this stage. They then started a new stage (Fig. 1c). In each trial, the images appeared on the grid, followed by the sound that started 1500 ms later. Another 2500 ms later, the image was removed from the screen and the circular grid turned 45° clockwise in a manner intended to mask the previous location. This rotation animation lasted 1000 ms and was immediately followed by a reappearance of the image at the center of the circular grid. The participant was then required to drag the image to its correct location and heard the sound again every time the image was picked up or dropped. Once this self-paced positioning was complete, the sound was played again and the image appeared in its true location (together with the user-estimated location and connected by an arrow) for 1500 ms. This feedback phase was followed by a 500-ms white-screen inter-trial interval. A total of 60 trials were presented in this stage, two for each image (order pseudorandomized to avoid consecutive repetitions of the same image).

The final step of the training phase consisted of a positioning exercise (Fig. 1d). Each image was positioned at the center of the circular grid. The associated sound was presented simultaneously. Participants were required to drag and drop the image and feedback was provided as described in the previous paragraph. A total of 30 trials were presented in this stage, one for each image (in random order). Throughout the different learning stages, no learning criterion was imposed and the number of exposures and positioning trials was equal for all three image groups.

As noted above, training blocks consisted of both TBR and TBF items in an interspersed manner to ensure that participant remained attentive when exposed to TBF items. Therefore, although instructed not to encode information regarding the TBF items, participants were still required to position them in their correct location (Fig. 1c,d). We did not instruct participants to use any specific strategy in order to complete the task without committing the locations to memory. However, as expected, encoding was different for TBF and TBR items, as evidenced by pre-sleep accuracy rates in the pre-nap memory test (see below). Importantly, any differences in encoding strategies between TBF and TBR items during training are inconsequential to our chief results, because our hypotheses concerned differences between the TBF-c and TBF-n conditions and not on any differences between these two conditions and the TBR condition.

Upon finishing the training phase, participants were offered a short break and were then outfitted with an EEG cap (described below). Next, the pre-nap memory test (T1; Fig. 1e) was administered. All 30 images, in addition to ten novel images, were presented in random order. For each image, the participant first had to specify whether they had seen the image before (“I’m sure it’s old”, “I think it’s old”, “I think it’s new”, “I’m sure it’s new”). If the image was rated as new (i.e., one of the latter two options was chosen), the test trial was concluded. Otherwise, the participant next had to specify which of the eight frame colors was associated with the image. Next, they used an on-screen scale or the mouse wheel to specify the size of the image. Then, the image was shown in the middle of the circular grid and the participant attempted to drag and drop it in its correct location. Finally, participants were asked to specify whether the image was TBF or TBR (but were not required to state which specific sound it was associated with). Throughout this phase, no feedback was provided and no sounds were presented. Participants were explicitly instructed to try their best during this test, even for images that they were not to commit to memory.

After completing T1, participants were given a 90-minute nap opportunity. The futon that was used as a chair within the experimental chamber was converted to a bed, with sheets and a pillow provided. The lights were turned off and pink noise at an intensity of 47 dB SPL was continuously presented. Once the participant reached SWS, the sound associated with the TBF-c group was repeatedly presented at an average intensity of 51 dB SPL (stimulus onset asynchrony = 5000 ms). Sounds were immediately discontinued upon detection of arousal or transition to another sleep stage, and in cases when SWS was not reached within 45 min, TMR was initiated during stage 2 of sleep.

After 90 minutes, participants were woken and were allowed to freshen up and clean the conductive gel from their hair and faces. The futon was converted back into a chair. Participants then began another test phase (T2; Fig. 1e), which was identical to T1 with the exception that a new set of ten novel images was used instead of the set used in T1. After the test, participants were tested on image-sound associations. First, they were exposed to each sound (in a random order), and for each one they were allowed 3 min to type in a brief description of as many associated images as they could recall (one participant had only 1 minute due to a technical error). These descriptions were manually associated with the images during offline analysis. Next, participants were similarly tested using a recognition test, which was identical to the one used during the first phase of the study, only without feedback. Once they completed these tests, participants were debriefed and dismissed.

### Electroencephalography and polysomnography

EEG was recorded using Ag/AgCl active electrodes (Biosemi ActiveTwo, Amsterdam). In addition to 32 scalp electrodes, contacts were placed on the mastoids, next to the eyes, and on the chin. All recordings were made at 512 Hz. Data was re-referenced offline using the average of both mastoid electrodes. Sleep scoring was based on the guidelines published by the American Academy of Sleep Medicine^36^ and was approximated online (while the participant was sleeping) and completed in full offline using EEGLAB^37^ and sleepSMG (http://sleepsmg.sourceforge.net) packages for Matlab (The MathWorks Inc, Natick, MA). Offline scoring was done by two independent raters, both of whom were not privy to when sounds were presented. Any discrepancies were subsequently reconciled by one of the two raters.

### Analysis

Analyses consisted of repeated-measures ANOVAs, two-tailed one-sample t-tests, and one-tailed paired t-tests. One-tailed paired tests were only used when testing the main directional hypothesis (i.e., that TMR will weaken memory). Since our study does not use individual sounds per image, any effects of TMR depend on the arbitrary association between cued sounds and images. Therefore, analyses were conducted both on the full dataset, and also using only the subset of images that were correctly associated with the one correct sound in the final image-sound-association recall test.

Differences between the effects of sleep on TBF-c and TBF-n items may have been due differences in the initial, pre-sleep error rates. We used both the full dataset and the one using the correctly associated items to rule this out confounding factor (Supplementary Fig. 2). To test whether effects would still hold for datasets in which smaller pre-sleep differences existed, we repeatedly subsampled our data in the following manner: for each participant and each of the two TBF conditions, we removed data for a single image at random, and then calculated the new average error rates per condition and participant. This was repeated 10^5^ times and the *p*-value for the pre-sleep difference across participants was calculated for each repetition. For sets with *p*-values over 0.3, 0.4 and 0.5, we tested the TMR effect (i.e., the difference between sleep-related effects for the two TBF conditions). Supplementary Fig. 2 shows the histogram of the *p*-values of this effect using the three cutoff values, showing that even when pre-sleep differences were eliminated, TMR still weakened memory.

## Supplementary information

Supplementary Materials.

## Data Availability

The datasets generated during the current study are available from the corresponding author on reasonable request.
