# Author Correction: Targeted memory reactivation during sleep boosts intentional forgetting of spatial locations

**DOI:** 10.1038/s41598-020-61784-8

**Published:** 2020-03-10

**Authors:** Eitan Schechtman, Sarah Witkowski, Anna Lampe, Brianna J. Wilson, Ken A. Paller

**Affiliations:** Department of Psychology, Northwestern University, Evanston, IL 60208 USA

Correction to: *Scientific Reports* 10.1038/s41598-020-59019-x, published online 11 February 2020

The Acknowledgements section in this Article is incomplete.

“This work was supported by NSF grants BCS-1461088 and BCS-1921678. E.S. is a funded by the Human Frontier Science Program and the Zuckerman STEM Leadership Program.”

should read:

“This work was supported by NSF grants BCS-1461088 and BCS-1921678 and by the Mind Science Foundation. E.S. is funded by the Human Frontier Science Program and the Zuckerman STEM Leadership Program.”

