# Supplementary material for: Targeted memory reactivation during sleep boosts intentional forgetting of spatial locations
Source: Sci Rep. 2020 Feb 11;10:2327. doi: 10.1038/s41598-020-59019-x (PMC7012837; doi:10.1038/s41598-020-59019-x)
Supplement: Supplementary file 1 — Supplementary Materials. [file 41598_2020_59019_MOESM1_ESM.pdf]

Title: **Targeted memory reactivation during sleep boosts intentional forgetting of spatial locations**

Eitan Schechtman[1], Sarah Witkowski[1], Anna Lampe[1], Brianna J. Wilson[1] & Ken A. Paller[1]

Supplementary Materials include:

1. Supplementary Figure 1
2. Supplementary Figure 2
3. Supplementary Figure 3

<u>Supplementary Figure 1</u>: Training data shows no pre-sleep differences between TBR-c and TBR-n conditions. (a) During image-sound-association training (see Figure 1), participants had up to five attempts to reach the learning criterion. Differences between the number of attempts were not significant between groups [Repeated-Measures ANOVA, $F(29,2)=0.01$, $p=0.99$]. (b) During image-location learning, participant first had to position items in a guided manner (Figure 1c). This part of training repeated twice and there was a trend towards an improvement between the first and second run [Repeated-Measures ANOVA, $F(29,1)=3.55$, $p=0.07$]. However, there was no difference between the TBF-c and TBF-n groups [$F(29,1)=0.003$, $p=0.95$] and no significant interaction between run and group [$F(29,1)=0.09$, $p=0.77$]. (c) During image-location training, participants then had to position the items without guidance (Figure 1d). Their performance for items associated with the TBF-c and TBF-n groups was not significantly different [$t(29)=0.57$, $p=0.57$].

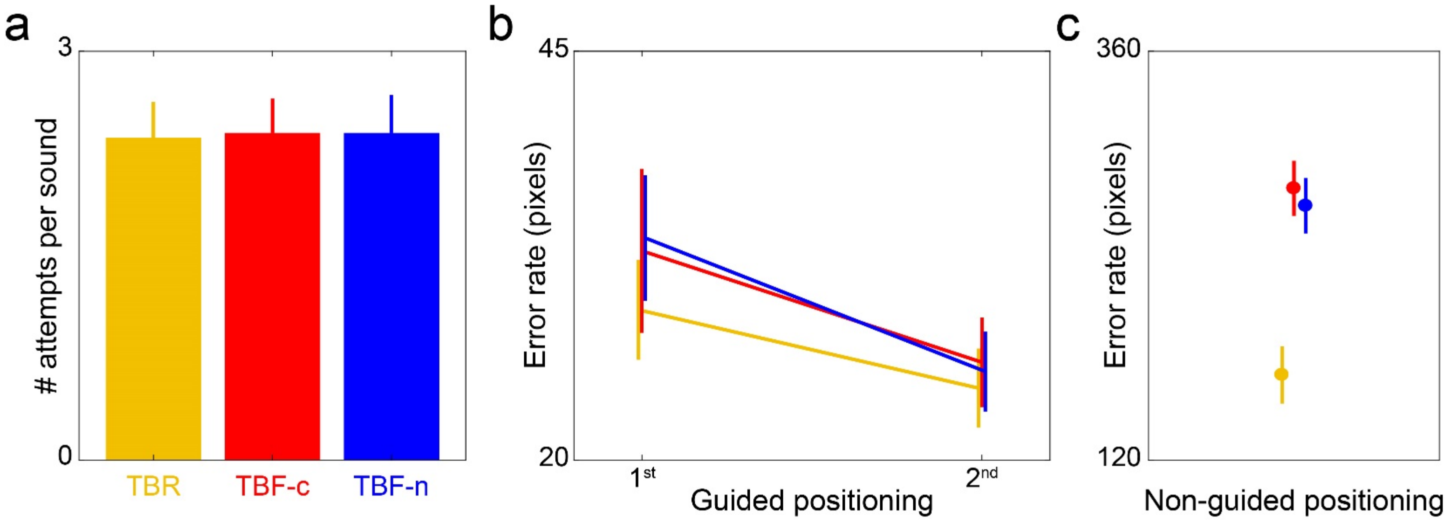

<u>Supplementary Figure 2</u>: An examination of pre-sleep differences between group and their possible influence on TMR. To evaluate pre-sleep differences, we subsampled the data (see Methods) and then selected subsampled datasets in which pre-sleep between-group differences had a significance level of $p \geq 0.5$ (light gray), $p \geq 0.4$ (medium shade of gray) or $p \geq 0.3$ (dark gray). For each group we created a histogram of *p*-values for the critical TMR effect (i.e., the difference between TBF-c and TBF-n sleep-related differences). This analysis was conducted twice – once for all items, to create the top panel, and once for the recalled items, to create the bottom panel.

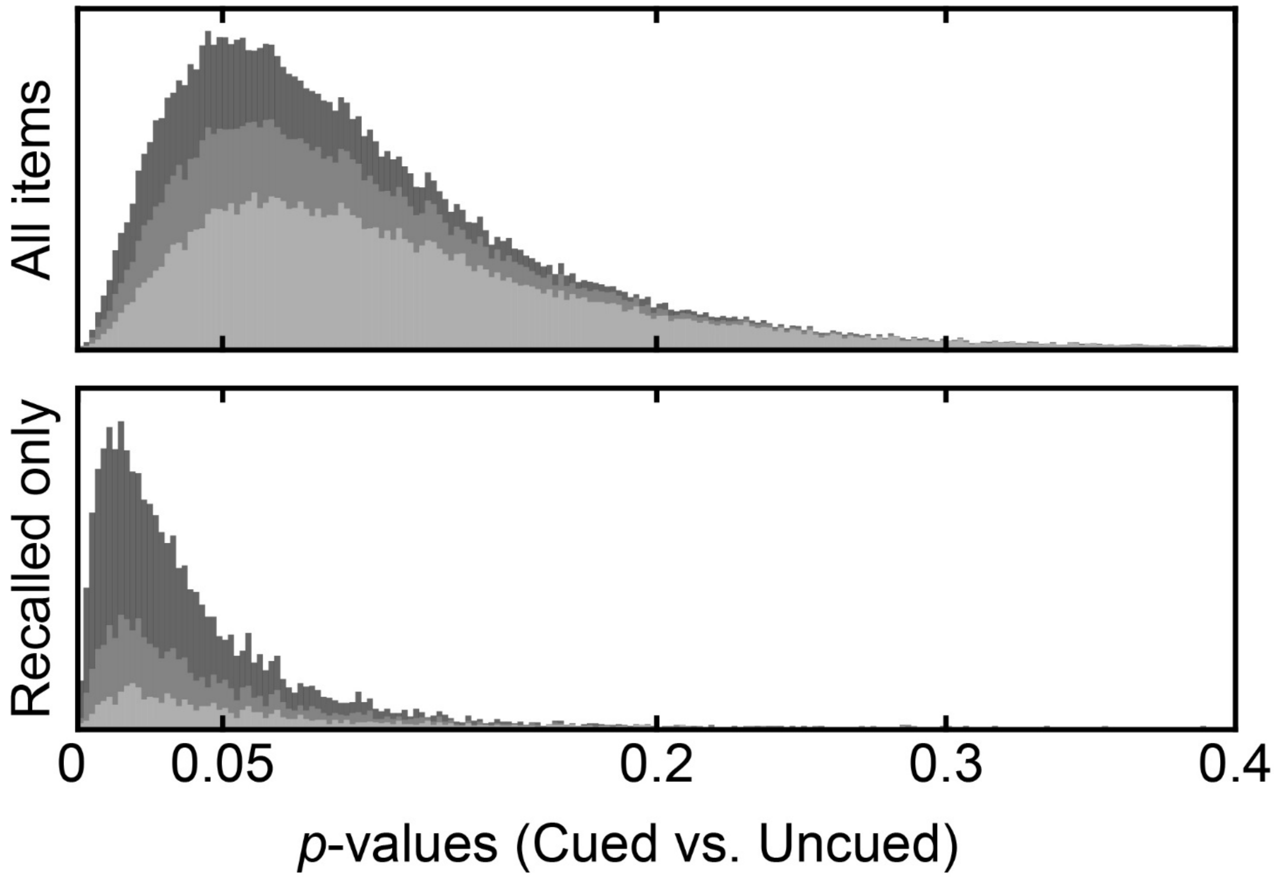

__Supplementary Figure 3__**: Memory for features other than spatial memory, based on pilot data and data from the experiment.** (a-d) Pilot data was collected for 10 participants. The task was intended to assess directed forgetting in waking and was identical to the one depicted in Figure 1a, up until the pre-sleep test (T1). Additionally, 20 items were used for each of the three conditions instead of 10. Results are shown for T1 for the TBR and TBF conditions (pooling the TBF items associated with both sounds together). (a) The percentage of previously learned items recognized as "sure it's old", "think it's old", "think it's new", "sure it's new" (i.e., old-new categorization) was not significantly different between TBR and TBF conditions. (b) The forgetting instructions significantly reduced memory for the spatial location of the TBF items relative to TBR items. (c) The percent of frame colors successfully recognized was not significantly different between TBR and TBF conditions. (d) Memory for the size of the side of the square image was not significantly different between TBR and TBF conditions. (e-h) Memory for all four measured features for the experimental dataset. Data are shown for T1 and pooled across difference TBF groups, as described for (a-d). Note that some differences which were not significant for the pilot data were significant for the experimental data, possibly due to the larger data set or due to fewer items per group in the final experimental design. (e) Old-new categorization was not significantly different between TBR and TBF conditions. (f) The forgetting instructions significantly reduced memory for the spatial location of the TBF items relative to TBR items. (c) The forgetting instructions significantly reduced memory for frame color recognition. (d) The forgetting instructions significantly reduced memory for frame size. (i-k) Analysis of the effects of sleep (T1/T2) and cuing (TBF-c/TBF-n) on memory of features other than location. (i) The change in the old-new categorization between T1 and T2 was not significantly different between the TBF-n and TBF-c conditions. (j) The change in frame color memory between T1 and T2 was not significantly different between the TBF-n and TBF-c conditions. (k) The change in frame size memory between T1 and T2 was not significantly different between the TBF-n and TBF-c conditions.

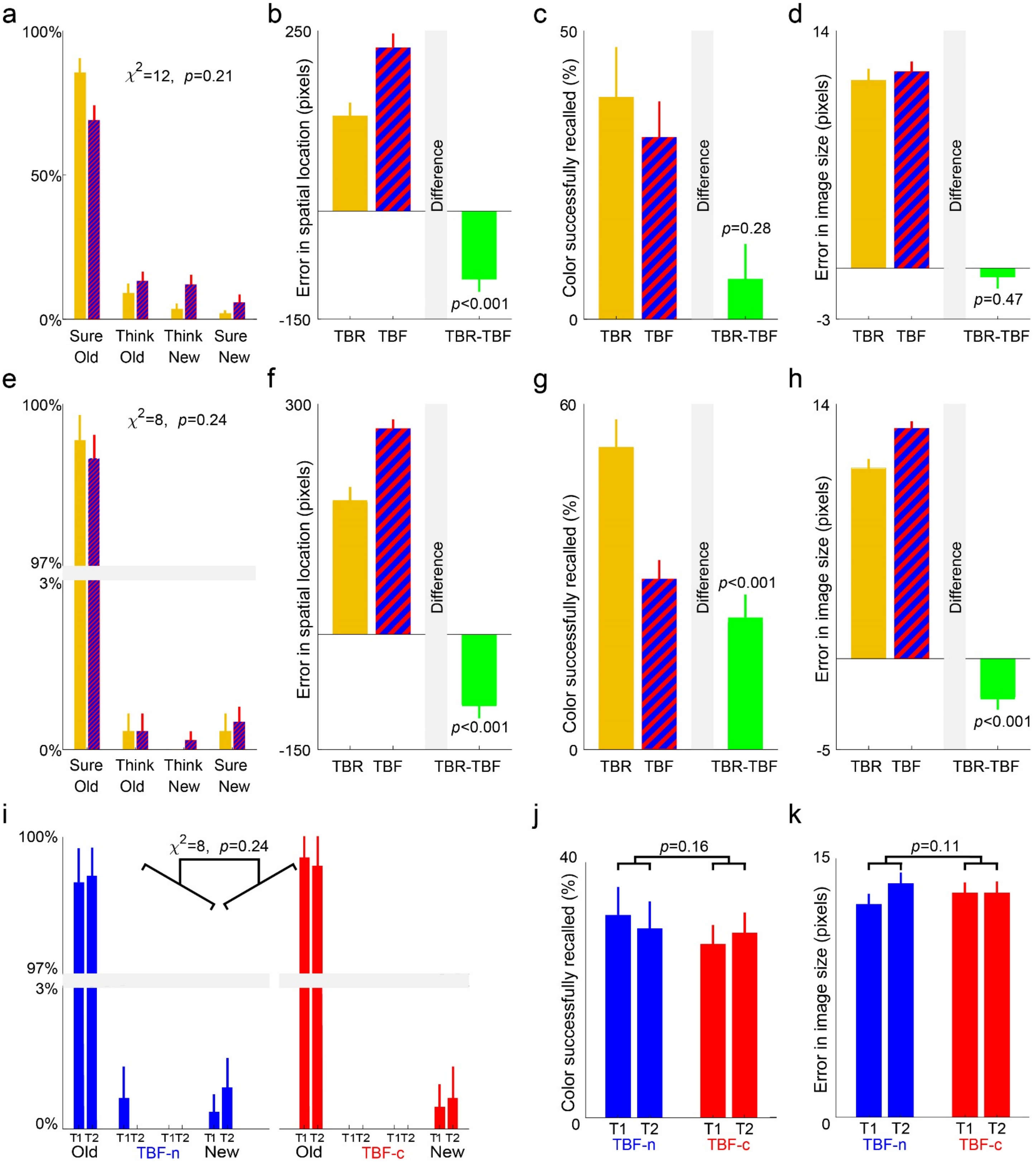
a
100%
50%
0%
χ^2=12, p=0.21
Sure Old
Think Old
Think New
Sure New
b
250
-150
Error in spatial location (pixels)
Difference
p<0.001
TBR
TBF
TBR-TBF
c
50
0
Color successfully recalled (%)
Difference
p=0.28
TBR
TBF
TBR-TBF
d
14
-3
Error in image size (pixels)
Difference
p=0.47
TBR
TBF
TBR-TBF
e
100%
97%
3%
0%
χ^2=8, p=0.24
Sure Old
Think Old
Think New
Sure New
f
300
-150
Error in spatial location (pixels)
Difference
p<0.001
TBR
TBF
TBR-TBF
g
60
0
Color successfully recalled (%)
Difference
p<0.001
TBR
TBF
TBR-TBF
h
14
-5
Error in image size (pixels)
Difference
p<0.001
TBR
TBF
TBR-TBF
i
100%
97%
3%
0%
χ^2=8, p=0.24
T1 T2
Old
TBF-n
New
Old
TBF-c
New
j
40
0
p=0.16
Color successfully recalled (%)
T1 T2
TBF-n
TBF-c
k
15
0
p=0.11
Error in image size (pixels)
T1 T2
TBF-n
TBF-c
